# Accurate detection of heart rate using in-ear photoplethysmography in a clinical setting

**DOI:** 10.3389/fdgth.2022.909519

**Published:** 2022-08-17

**Authors:** Tim Adams, Sophie Wagner, Melanie Baldinger, Incinur Zellhuber, Michael Weber, Daniel Nass, Rainer Surges

**Affiliations:** ^^1^^ Cosinuss GmbH, Munich, Germany; ^^2^^ Associate Professorship of Sport Equipment and Sport Materials, Technical University of Munich, Munich, Germany; ^^3^^ Department of Epileptology, University Hospital Bonn, Bonn, Germany

**Keywords:** validation, heart rate, photoplethysmography, in-ear, wearable sensor, continuous monitoring, epilepsy

## Abstract

**Background:**

Recent research has shown that photoplethysmography (PPG) based wearable sensors offer a promising potential for chronic disease monitoring. The aim of the present study was to assess the performance of an in-ear wearable PPG sensor in acquiring valid and reliable heart rate measurements in a clinical setting, with epileptic patients.

**Methods:**

Patients undergoing video-electroencephalography (EEG) monitoring with concomitant one-lead electrocardiographic (ECG) recordings were equipped with an in-ear sensor developed by cosinuss°.

**Results:**

In total, 2,048 h of recording from 97 patients with simultaneous ECG and in-ear heart rate data were included in the analysis. The comparison of the quality-filtered in-ear heart rate data with the reference ECG resulted in a bias of 0.78 bpm with a standard deviation of ±2.54 bpm; Pearson’s Correlation Coefficient PCC = 0.83; Intraclass Correlation Coefficient ICC = 0.81 and mean absolute percentage error MAPE = 2.57.

**Conclusion:**

These data confirm that the in-ear wearable PPG sensor provides accurate heart rate measurements in comparison with ECG under realistic clinical conditions, especially with a signal quality indicator. Further research is required to investigate whether this technology is helpful in identifying seizure-related cardiovascular changes.

## 1. Introduction

Current advancements in optical wearable sensors have made them an attractive tool in disease monitoring, in which patients’ physiological and behavioral metrics are captured in a continuous, unobtrusive way (1). A notable percentage of the optical sensing devices use the PPG, a non-invasive technology, measuring optical variations based on blood volume changes (2). Heart rate is one of the key neurophysiological metrics when monitoring patients’ condition. In PPG measurements a certain proportion of the emitted light is absorbed through tissue. Since tissue absorption depends on the local blood volume, it is possible to infer the heart rate from the ratio of absorbed to reflected light, which is measured by a photodiode.

In a medical setting, heart rate is typically measured by an ECG, involving electrodes on the skin. This method is uncomfortable for the patient, not suitable for continuous, long-term monitoring, especially when patients move. PPG-based heart rate monitors can be a promising alternative to ECG. The comparison of PPG and ECG in heart rate monitoring has already been discussed in a number of studies. An example of raw signals from a PPG sensor and an ECG is provided in **Supplementary Materials, Figure S1**. Some studies comparing the two methods found differences, other groups reported an overall strong agreement (for an overview see (3)). Nevertheless, these studies had diverse experimental settings, measurement locations and/or methods of analysis. Results on the influence of the sensor location are sparse (see for example (4)). Ear-worn PPG devices have been discussed in several studies as an alternative to ECG (5–7). When the sensor was placed on the earlobe, moderate to good accordance between PPG and ECG was shown (8–10). Overall, previous studies have shown that the PPG is a promising technology regarding its use in ear-worn wearables. However, most of the studies mentioned evaluated non-commercial, prototype ear-worn devices (11, 12).

Recent research has shown that alterations in heart rate can be used as a biomarker in epilepsy monitoring (13–16). Epilepsy is a common neurological disease affecting about 0.6% of the world’s population (17). It is characterized by recurrent seizures with highly variable symptoms, ranging from mild pure body sensations to severe symptoms such as loss of body movements control and consciousness (18). A significant proportion of seizures are not recalled by patients for various reasons, hampering the accurate documentation of disease activity by seizure diaries held by patients (19). Therefore novel treatment options allowing real-time seizure monitoring in daily life can benefit patients tremendously by reducing unpredictability and improving their quality of life (20, 21). In this context, epilepsy monitoring is one of the potential medical applications of in-ear wearable PPG sensors.

Within the scope of the EPItect project (22), we hypothesize that PPG-based sensors placed inside the ear canal are well protected from movement-related artifacts and have a potential to accurately capture all necessary biosignals that are required to detect a wide range of epileptic seizures. As a fundamental step towards this goal, here we aimed to evaluate the performance of an in-ear PPG-based wearable sensor in acquiring accurate and reliable heart rate with respect to an ECG. This CE-marked device, cosinuss° One (developed by Cosinuss GmbH, Munich, Germany), is capable of detecting heart rate by using PPG signals, head movements by measuring acceleration in three dimensions and core body temperature via contact temperature sensor.

Our results demonstrate that the cosinuss° One with PPG-technology can derive heart rate with high accuracy in a real-life clinical setting, in an epileptic patient population. Our findings also indicate the necessity of a signal qualifier for identifying medically useful data points. To our knowledge, this study is the first to conduct a detailed validation of a commercial in-ear heart rate monitor using PPG in a clinical setting.

## 2. Materials and methods

### 2.1. Subjects

Patients (≥18 years) with refractory epilepsy, who underwent video-EEG monitoring in the Department of Epileptology in University of Bonn Medical Center, for non-invasive presurgical evaluation, syndrome diagnosis, or monitoring of seizure control, were included in the study. Informed consent was obtained from all subjects involved in the study. Subjects participated in long-term VEEG-monitoring for 1–13 days depending on the individual diagnosis and the amount of seizures recorded. During that time, videos of the subjects were recorded as well as the EEG, pulse oximetry, and one-lead ECG. Additionally, the patients wore the cosinuss° One in-ear sensor, which recorded their heart rate, PPG-signals, core body temperature and the 3D-acceleration data. Every six hours the study nurses changed the in-ear sensor to a fully charged device. The study was approved by the local medical ethics committee (Ethikkommission der Medizinischen Fakultät der Rheinischen Friedrich-Wilhelms-Universität Bonn, No. 355/16) and conducted in accordance with the Declaration of Helsinki.

### 2.2. In-ear sensor: Cosinuss° One

**Figure 1:**
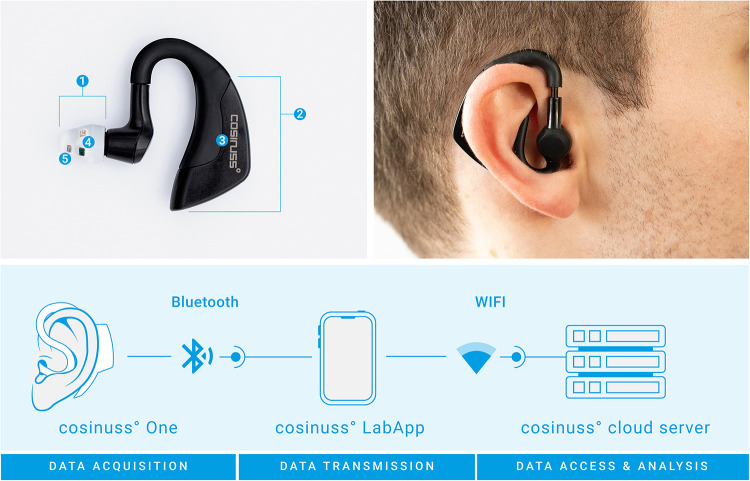
Concept of the cosinuss° One in-ear sensor used as a wearable to detect heart rate in the study. Top left image: (1) sensor head, (2) processing unit, (3) accelerometer, (4) temperature sensor, (5) PPG sensor elements. Bottom image: Acquired data are sent to the cosinuss° LabApp smartphone application via Bluetooth, which are then automatically uploaded to the cloud via a Wi-Fi connection.

To record the PPG signals, we used the in-ear sensor cosinuss° One (Cosinuss GmbH, Munich, Germany), which is shown in Figure 1. It is a mobile wearable sensor that continuously measures the core body temperature, heart rate and acceleration. In addition, it records the intervals between heart beats (known as RR intervals), fluctuations of which is correlated with heart rate variability. The cosinuss° One incorporates a PPG sensor element (combining a light-emitting diode (LED) and a photodiode), a resistance temperature sensor and a 3D-accelerometer. For heart rate measurements the so-called circummission method uses green light, which is emitted into the ear canal by a LED (23). The photodiode measures the proportion of the light reflected. The heart rate is automatically calculated by the sensor device and can directly be derived from the data output.

The sensor has a size of (H) 45 × (W) 38 × (D) 18 mm and a weight of 6.5 g. Close contact of the sensor with the tissue inside the ear canal is crucial for a precise measurement. For this reason, the LED, photodiode and temperature sensor are placed on a soft silicone shield that fits firmly into the ear canal. The sensor head is available in three sizes (S, M, L) to adapt to different ear canal sizes of children, adults and elderly. The processing unit is behind the ear. Battery life of the sensor is at least 7 h according to the manufacturer (24). The cosinuss° One is certified with the consumer CE-mark.

### 2.3. Data collection

The one-lead ECG was recorded at lead I with a sampling rate of 256 Hz, using the Micromed system (Micromed S.p.A., Treviso, Italy). The data collected from the in-ear sensor was recorded in real-time with a sampling rate of 50 Hz for the PPG and acceleration data, and approximately 1 Hz for the vital parameters (heart rate with RR intervals, core body temperature). The data were continuously transferred from the sensor to an Android smartphone using Bluetooth Low Energy (Bluetooth 4.2), without compression. From the Android device the data was automatically sent to the cosinuss° cloud server via a Wi-Fi connection for further analysis and archiving of the data using the cosinuss° LabApp (see Figure 1). All personal identifying information and medical health data on the cosinuss° cloud server are pseudonymized. Only authorized persons have access to the data.

### 2.4. Data analysis

Data was analyzed using Python 3. Heart rate data from the in-ear sensor is directly derived from the cosinuss° cloud server. Data from the ECG recording was exported and uploaded to the same cloud server by the clinical staff. For the ECG data the R-wave peaks are detected using the well-approved Pan-Tompkins-algorithm (25). From the resulting RR-intervals, the heart rate values in units of beats per minute (bpm) are obtained.

#### 2.4.1. Data processing & quality filtering

The in-ear sensor provides a signal quality indicator (QI, ranging from 0–100 (a.u.), with 100 being the most reliable). It is calculated at each heart rate data point and indicates its reliability by measuring the PPG signal dominance in relation to the perturbations. Before the following steps in data processing, this quality measure is smoothed and then used to remove data points that do not exceed a specified quality threshold. With the choice of this threshold, a trade-off between improvement of statistical values and loss of data is made.

**Figure 2:**
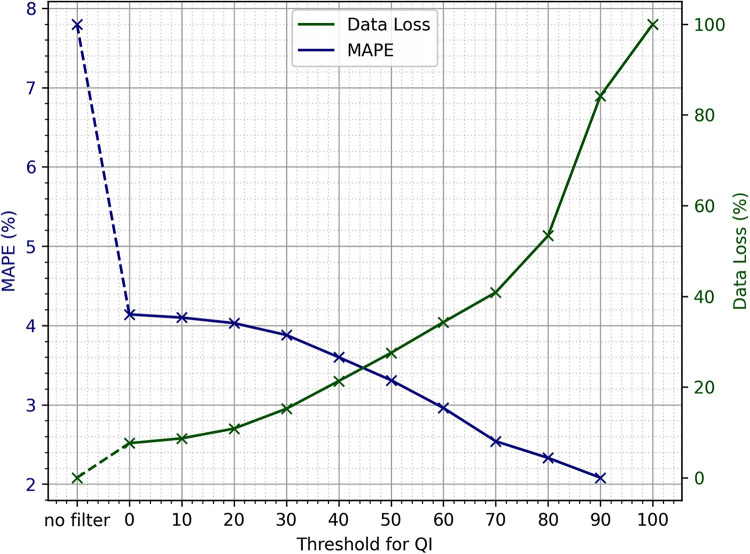
Sensitivity analysis of the quality indicator threshold, provided by the in-ear sensor to be used for quality filtering, in relation to mean absolute percentage error (MAPE) and data loss.

To determine the threshold QI value, a sensitivity analysis was performed. It was assessed how data loss and mean absolute percentage error (MAPE) changes with an increasing quality threshold. MAPE is computed as follows:

(1) $$\text{MAPE}=\frac{100\text{\%}}{n}\sum_{n=1}^{n}\left|\frac{A_{t}-F_{t}}{A_{t}}\right|$$

where $A_{t}$ is the reference value and $F_{t}$ is the value as measured by the in-ear device. How exactly these simultaneous data points $A_{t}$ and $F_{t}$ were extracted is explained in more detail in 2.4.2. The measure of data loss is obtained by comparing the absolute number of available raw data points before and after filtering for quality. The results can be seen in Figure 2. The higher the threshold, the more data will be removed by the quality filter, while the results of the statistical analysis performed on the remaining data improve. Using the smallest threshold, where the QI needs to be larger than 0, is already an improvement to performing no quality filtering at all. It is mainly because the QI is set to 0 for each data point where the QI’s x-value and the corresponding heart rate data point’s x-value differ by more than 2.5 s and when the heart rate is not within the range of 40 to 148 bpm. For thresholds 20 or larger, there is a near-linear relationship between the threshold of the quality indicator and percentage of data points that are removed based on this threshold, ranging from 11% data loss at a threshold of 20 to 53% data loss at a threshold of 80. For thresholds >80, the slope is slightly steeper, reaching 100% data loss at a threshold of 100. As expected, the MAPE drops with increasing QI threshold. Between thresholds of 40 and 80, the slope of decline is the steepest. Using a quality threshold of 0, the MAPE is at 4.14, and from a threshold of 70 upwards, the MAPE is below 2.6.

The sensitivity analysis shows that the choice of the exact threshold can be made at liberty, as the trade-off between data loss and MAPE is nearly linear if using thresholds of 80 and lower. If the primary goal of an evaluation is to include as much of the original data as possible, the smallest threshold would suffice to obtain reasonable heart rate values. Due to the large dataset available, it was decided that losing a larger portion of data in order to optimize for quality would still leave sufficient amounts of data for the analysis. As the data loss incline gets steeper upwards of a QI threshold of 80, and the MAPE is well below 3 upwards of 70, it was decided to use 70 as the QI threshold.

Only heart rate data points that exceeded the quality threshold were kept, removing about 40% of the data of the in-ear sensor. The main reason for this large percentage is the fact that the quality threshold discards long periods of measurement if the patient is not assigned a well-fitting sensor size or if the device is not worn properly in the ear canal. The reference ECG signal also underwent data processing in order to detect and remove unreliable data points due to motion artifacts and poor placement of the electrodes. A value was considered an outlier, and was discarded, if it deviated by more than 8 bpm from the median of itself and the 14 data points that follow it. If, within this area of 15 data points, more than three values were considered outliers, all 15 points were discarded. This step reduced the ECG data by 23%.

**Figure 3:**
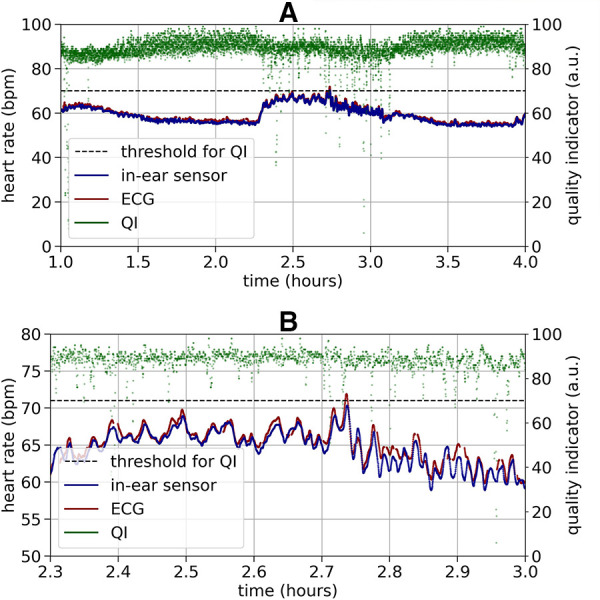
Top plot: Example of a high quality heart rate recording (bpm) over three hours by the ECG (red) and the in-ear sensor (blue). The signal quality indicator of the in-ear sensor (green) is above the quality threshold of 70 (indicated by the dashed line) at nearly all times of the recording. The heart rate data of the in-ear sensor and of the ECG overlap in a way that the red line of the ECG is completely hidden by the blue line of the in-ear sensor. Bottom plot: For better visibility this figure shows a short clipping of the recording above. During a period of relatively high variance in the heart rate, compared to the rest of the measurement, the heart rate values obtained by the ECG (red) and the in-ear sensor (blue) still show high agreement.

**Figure 4:**
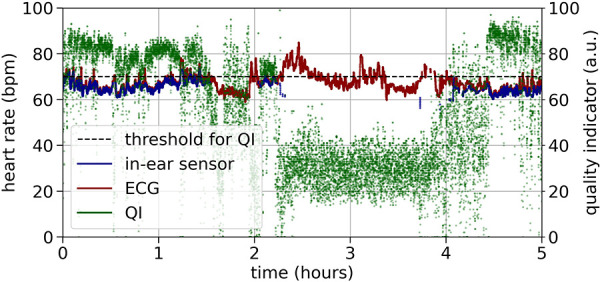
Example of a critical quality heart rate recording (bpm) over five hours by the ECG (red) and the in-ear sensor (blue). The signal quality indicator of the in-ear sensor (green) returns values below the quality threshold of 70 (indicated by the dashed line) for certain periods of time.

Figure 3 shows an example recording of heart rate over three hours. As a relatively high quality indicator predicts, the in-ear sensor coincides well with the ECG data. An enlarged segment of this example can be seen in Figure 3, bottom plot. In contrast, a recording that exhibits critically low quality values for a certain period of time is shown in Figure 4. The quality indicator (shown in green), while stable at first, drops below the critical threshold of 70 (indicated by the dashed line) after one hour, most likely because the sensor tip has lost its firm contact with the ear canal tissue. During this time, the in-ear sensor’s data points are dismissed due to their unreliability. Towards the end of the measurement, the quality rises above the threshold again, as the sensor may have gotten readjusted into the ear.

After data cleansing, the heart rate data of the ECG and the in-ear sensor were matched based on their respective time stamps, keeping only simultaneous data points of sufficient quality where signals from both sources were available.

#### 2.4.2. Statistical analysis

For further analysis, the data points were combined into time windows of two seconds. The average value of each time bin as provided by the in-ear sensor is compared to the respective average value of the ECG recording, testing both precision and reliability. Correlation between recordings was measured using Pearson’s correlation coefficient (PCC). Since high correlation does not necessarily guarantee high agreement, the Intraclass Correlation Coefficient—ICC (3,1) as defined by Shrout and Fleiss (26)—is considered to be a more suitable measure to quantify an instrument’s accuracy (27). Besides the ICC, MAPE was computed to assess accuracy. To test precision, Bland-Altman plots were employed, which plot the difference between two samples over their mean value (28). Thus, it shows the accordance of the two measurements, while also visualizing whether a measurement error is systematic, i.e. correlated with the value it is measuring. The mean value over all deviations within a recording session leads to an absolute bias of the two measurement methods and the lower (LLOA) and upper (ULOA) limits of agreement can be derived using the standard deviation (SD): LLOA=bias−1.96×SD and ULOA=bias+1.96×SD, respectively. For the following evaluation, the parameters deduced from the Bland-Altman plots (LLOA, ULOA, mean, standard deviation) were taken from a conjoined Bland-Altman plot of all data points of each person. PCC, R-squared, ICC, and MAPE were calculated for each measurement individually and then averaged per person.

## 3. Results

### 3.1. Study characteristics

A total of 174 subjects (age: 43.2±17.7 years; gender: male 46%, female 54%) participated in this study. At the beginning of the study a prototype in-ear sensor with a red and infrared LED was used. The reason for that was to be able to derive more physiological signs (such as the blood oxygen saturation) from the PPG signal. However, during the recordings with the first 65 patients, we observed substantial motion artifacts, which led to unreliable heart rate measurements. Since the PPG signals from green LED are more robust in dealing with motion artifacts, after the first 65 patients, we decided to switch to the cosinuss° One in-ear sensor using a green LED, to acquire more reliable heart rate measurements. Only data recorded with the cosinuss° One was taken into account for further analysis. During the initial trial a few recordings of the in-ear sensor were missed due to technical issues. Furthermore, not all ECG data was stored permanently for the first half of the study, where only the first 12–24 h recording per patient was stored. This results in a total of 97 subjects and 2048.46 h of recording including ictal as well as interictal activity.

After the quality filtering and smoothing of the simultaneous ECG and PPG data -as described in Section D, the heart rate data from the in-ear sensor and the ECG amount to 1396.77 h of recording. Using recordings from 97 different patients, this leads to an average of 14.4 h of data per patient.

### 3.2. Effect of quality filtering on in-ear heart rate measurements

**Figure 5:**
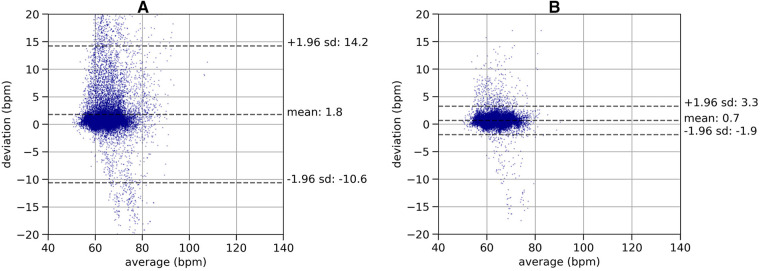
Plot (**A**): Bland-Altman plot of a recording of heart rate without quality filtering, comparing the in-ear sensor’s heart rate measurement with the ECG over a recording time of 63 h. The plot shows the deviations of the heart rate values (y-axis) relative to the mean of the two methods (x-axis). The dashed lines indicate the mean value of the deviations (bias) and the lower (LLOA) and upper (ULOA) limits of agreement as calculated as bias±1.96×SD. This leads to a bias of 1.8 bpm and limits of agreement of LLOA=−10.6 bpm and ULOA=14.2 bpm. Plot (**B**): Bland-Altman plot of a recording of heart rate with quality filtering, comparing the in-ear sensor’s heart rate measurement with the ECG over a recording time of 46 h. The same data set as in left plot is shown, removing the data points below the quality threshold. The lower (LLOA) and upper (ULOA) limits of agreement as calculated as bias±1.96×SD. Removing low quality data points leads to a bias of 0.7 bpm and limits of agreement of LLOA=−1.9 bpm and ULOA=3.3 bpm.

To analyze the effectiveness of the quality indicator on the in-ear heart rate measurement, we used Bland-Altman plots (28) and visualized the deviations of heart rate data from the in-ear sensor and the ECG, as shown in Figure 5. Data shown in Figure 5, subplot A), comprises a recording time of approximately 63 h. All data points originating from the in-ear sensor were used without filtering. There are some significant deviations of the in-ear sensor’s heart rate measurements to the ones of the ECG. Most deviations occur around a heart rate of 60–70 bpm, because the majority of this patient’s heartbeats were in this range. Said deviations are mostly positive, meaning that the ECG generally returned higher values than the in-ear sensor. The mean bias and standard deviation lead to a lower and upper limit of agreement of LLOA=−10.6 bpm and ULOA=14.2 bpm for this patient. Figure 5, subplot B), shows the same patient’s data; however, the in-ear sensor’s quality indicator was taken into consideration, removing all points that were below the threshold of 70, leaving 46 h of data. As a result, the upper limit of agreement improves to ULOA=3.3 bpm and the lower limit rises to LLOA=−1.9 bpm.

### 3.3. Accuracy of quality filtered in-ear heart rate measurements

**Figure 6:**
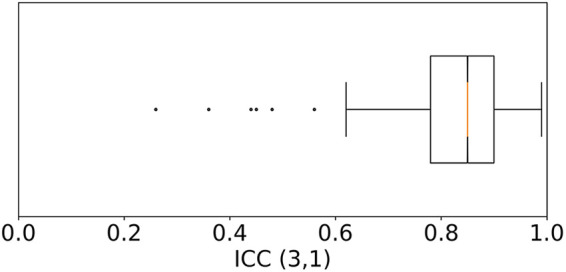
Box plot of the Intraclass Correlation Coefficient—ICC (3,1) as defined by Shrout and Fleiss (26)—comparing the quality filtered in-ear heart rate data to the heart rate from ECG over all 97 patients. Each recording was weighted by its duration. The median is at 0.85. 50% of the data points lie within the range of 0.78–0.9. Six outliers that score lower than 0.62 were identified.

After quality-filtering of all in-ear heart rate measurements, the comparison of the in-ear sensor and the ECG leads to a bias of 0.78 bpm and a standard deviation of 2.54 bpm. Table I visualizes the effectiveness of the quality filter by comparing statistical results using all data points to statistical results keeping only points of sufficient quality. The overall limits of agreement amounted to LLOA=−3.95 bpm and ULOA=5.53 bpm; the average ICC=0.81; while the average PCC scored slightly higher, at 0.84 over all recordings; the average MAPE was 2.43%. Removing unreliable points significantly improves all statistical parameters. As an example, Figure 6 shows a box plot of the ICC value over all measurements.

**Table I.** The resulting statistical values averaged over all 97 patients. Values are average duration, bias, standard deviation (SD), lower limit of agreement (LLOA), upper limit of agreement (ULOA), Intraclass Correlation Coefficient (ICC), Pearson’s Correlation Coefficient (PCC), the coefficient of determination, *R*^2^, in this case the squared Pearson’s correlation coefficient, and the mean absolute percentage error (MAPE).

	All data points	Quality-filtered
Duration (h)	21.12	14.40
Bias (bpm)	1.64	0.78
SD (bpm)	12.75	2.54
LLOA (bpm)	–23.35	–4.19
ULOA (bpm)	26.62	5.75
ICC	0.46 [0.41,0.51]	0.81 [0.78,0.84]
PCC	0.50 [0.45,0.55]	0.83 [0.80,0.86]
$R^{2}$	0.30 [0.25,0.35]	0.71 [0.68,0.75]
MAPE (%)	7.94 [6.42,9.46]	2.57 [2.24,2.90]

**Note**: The 95% confidence interval of the averaged ICC, PCC, $R^{2}$, and MAPE values are given in square brackets.

## 4. Discussion

The present study examined the accuracy of the cosinuss° One, an in-ear wearable sensor utilizing PPG-technology, for measuring heart rate in a real-life clinical setting, in comparison with ECG. The results prove that the in-ear PPG sensor can provide a valid heart rate measurement if further improved by filtering algorithms. Though achieving moderate results using all data points, applying the quality indicator of the in-ear sensor improved all statistical parameters by removing unreliable data points. If less data loss is desired, the quality threshold can be decreased without disproportionally increasing the MAPE, as both parameters have a near linear relationship to the threshold.

PCC and ICC were calculated to test the correlation of the in-ear sensor with the reference ECG device. The fact that the PCC (0.83) scored slightly higher than the ICC (0.81) is not surprising, as the in-ear sensor cosinuss° One shows a more or less constant bias of +0.8 bpm, and the PCC measure returns high correlation for linear relationships between recordings. In medicine, there is no consensus whether the ICC is a sufficient measure of quality for a heart rate measurement device. According to Rosner’s definition (29), the value obtained by the cosinuss° One in the present study signifies an excellent agreement, while according to Koo et al. (27), it signifies a good agreement. Fokkema et al. (30) suggest to interpret ICC values as excellent, good, moderate, and low agreement thresholds, if they are ≥0.90; 0.75–0.90; 0.60–0.75; and ≤0.60, respectively. This interpretation of ICC values indicates a good agreement between the cosinuss° One and the ECG recordings in this case.

Testing the accuracy of the in-ear sensor using MAPE resulted in 2.57%. There is no standard cut-off value for acceptable MAPEs, however Nelson et al. (31) consider a MAPE ≥10% as an indicator of inaccuracy, whereas Fokkema et al. (30) suggest a threshold of ≤5% for a value to be considered accurate. Etiwy et al. (32) tested the accuracy of four PPG-based wrist-worn heart rate measurement devices as well as the Polar H7 chest strap, comparing all to standard ECG limb leads. The devices were tested on 80 patients in cardiac rehabilitation while they were at rest, on a treadmill and on a stationary cycle. The Polar strap achieved the best results in all disciplines, reaching a MAPE of 0.9±1.6% during the resting period. The other wearable devices reached MAPEs between 4.1±7.2% (Apple watch on the stationary cycle) and 13±18% (Garmin device on the stationary cycle). Therefore, applying the previous interpretations of the MAPE in similar contexts, it can be concluded that the MAPE achieved by the in-ear sensor cosinuss° One in here (MAPE=2.57%) is comparably low and can be considered as accurate.

Bland-Altman analysis revealed that the cosinuss° One had a bias of +0.78 bpm and 95% of differences fall within +5.75 and −4.19 bpm of the ECG (ULOA - LLOA = 9.94 bpm). As the differences were calculated by subtracting the in-ear sensor’s value from the one of ECG’s and the mean bias is positive, we conclude that the in-ear sensor tends to slightly underestimate heart rate values. This result is in line with the findings of Passler et al. (6). In this previous work by Passler et al., the cosinuss° One in-ear sensor was compared and validated against an ECG. However, this validation was done on 20 healthy subjects during graded cycling, under laboratory conditions. Results of the in-ear sensor showed a bias of +0.4 bpm and 95% of all differences within +4.38 and −5.17 bpm of the ECG (ULOA - LLOA = 9.55 bpm) during resting conditions with a heart rate ≤90‘bpm. Furthermore the study demonstrated an excellent agreement (ICC=0.94; MAPE=2.5%) between the cosinuss° One and ECG recordings during resting conditions (mean heart rate ± standard deviation of 53.6±8.3 bpm as measured by the cosinuss° One) and a good agreement (ICC=0.84) at maximum heart rate (mean heart rate ± standard deviation of 181.6±6.4 bpm as measured by the cosinuss° One). There is no indication that the quality indicator of the in-ear sensor was taken into account. Therefore, we argue that the quality filtering algorithm of the cosinuss° One significantly improves the accuracy of the HR measurements, especially when applied in realistic clinical settings where a certain contingent of application errors must be assumed.

There are however some limitations to the performed study that could be addressed in future research. First, the study focused on the heart rate recordings of stationary patients, who were at resting-state. This leads to recordings of mostly moderate heart rate values and small changes. Therefore the accuracy of the in-ear sensor during periods of activity needs to be assessed in further work. Not yet understood is the more or less constant bias of 0.8 bpm on average below the ECG heart rate. Second, it must be noted that the current analysis did not take into account whether the patients also had cardiac diseases, which would affect the reproducibility of the results.

The third limitation of this study is that, while all patients experienced epileptic seizures during the recording periods, it was not assessed to what extent the seizure-related data was retained after quality filtering. We expect that the measurement quality deteriorates during seizures where muscles cramp and the heart rate increases. However, the validation of cosinuss° One against ECG is still a necessary prerequisite for future research on seizure detection. Taking into account the fact that the heart rate provided by the in-ear sensor proved to be reliable, a sudden deterioration in the quality may thus be used as a factor in seizure detection. While the loss of sensor-to-skin contact might be the main reason for decreased signal quality of the sensor, another one is seizure occurrence. If information is lost during seizures purely due to the event itself, the sensor will regain contact immediately afterwards, at which point the heart rate will be at a higher level and then gradually return to normal during the next minutes. This pattern, an example of which is shown in **Supplementary Figure S2**, could be detected and used for further analysis.

Furthermore, there are examples where the sensor’s signal quality stayed reasonably high during a seizure, as in **Supplementary Figure S2**, bottom plot, allowing for accurate heart rate measurements during the entire process.

The next step is to investigate the feasibility of using the heart rate, acceleration and PPG signals from the in-ear sensor to detect epileptic seizures. In a first attempt, Henze et al. (33) evaluated a multimodal approach by combining the 3D acceleration and heart rate data from the in-ear sensor developed by cosinuss°. They trained a selection of classification models to be used on the EPItect project dataset to detect tonic-clonic seizures. The labels originated from the project’s video-EEG recordings, which was annotated by medical professionals, to mark the on- and offset of seizures. This previous study found that using the combination of heart rate and acceleration data, in comparison to acceleration data only, indeed correlated with a higher number of tonic-clonic seizures. Bruno et al. (34) concluded in their systematic review on preictal heart rate changes that heart rate measurements could be valuable in identifying seizures prior to their apparent onset, although additional research is necessary to clearly mark down those patients that might benefit from heart rate monitoring. Furthermore, El Atrache et al. (35) identified changes in the PPG signal using wearable wristbands in the periictal periods of patients with focal impaired awareness seizures (FIAS) and concluded that FIAS detection using PPG data is feasible.

In the light of recent findings, we speculate that seizure detection will likely comprise a combination of features derived from multiple biosignals during, before and after the event. To make the in-ear optical sensor suitable for usage in epilepsy practice, future research should focus on the feasibility of using the heart rate, acceleration and PPG signals of the in-ear sensor to detect epileptic seizures and the impact of movement on the accuracy of the PPG-signal.

## 5. Conclusions

This validation study showed that the in-ear sensor cosinuss° One with PPG-technology can determine heart rate with high accuracy compared to single-lead ECG. The signal quality indicator of the sensor provides robust filtering of the unreliable PPG data, significantly increasing the overall accuracy of the heart rate measurements, which is critical in everyday clinical practice. This is the first paper to describe a detailed validation of heart rate derived from a commercial in-ear PPG sensor in a realistic clinical setting.

In the grand scheme of clinical epilepsy practice, our main question remains as whether in-ear PPG-based measurements can be used for epileptic seizure detection. Since epileptic patients may ultimately benefit from continuous, real-time monitoring and seizure detection using the in-ear sensor, establishing its accuracy in this specific setting was a crucial first step.

Subsequent studies should be conducted to explore whether the various biosignals measured by the wearable device might be useful to detect a broad spectrum of epileptic seizures.

## Data Availability

The raw data supporting the conclusions of this article will be made available by the authors upon qualified requests.
