# Supplementary material for: Accurate detection of heart rate using in-ear photoplethysmography in a clinical setting
Source: Front Digit Health. 2022 Aug 17;4:909519. doi: 10.3389/fdgth.2022.909519 (PMC9428405; doi:10.3389/fdgth.2022.909519)
Supplement: Supplementary file 1 [file Data_Sheet_1_v1.pdf]

Supplementary Figures

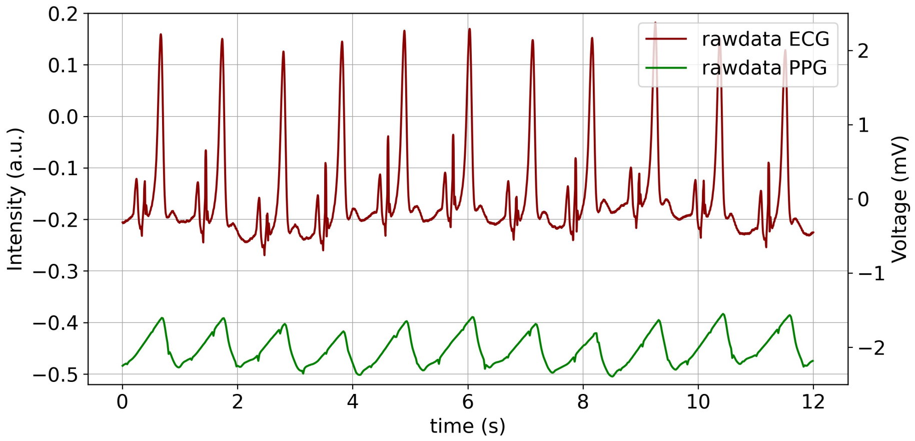

Figure S1. Comparison of the two types of raw signals that the heart rates were derived from: a PPG, provided by the in-ear sensor, and the same person's simultaneous ECG. For the in-ear sensor the graph looks very similar to pulse oximeters, which measure the heart rate using pulse waves within the vascular system and the oxygen saturation of the blood at a fingertip. In ECG, electrical signal of the heart is measured and typical voltage waveforms (called QRS complexes) for every heart beat is delivered. It must be noted that as both signals are raw signals, filtering and a heart rate algorithm are required to obtain a robust heart rate from either of them.

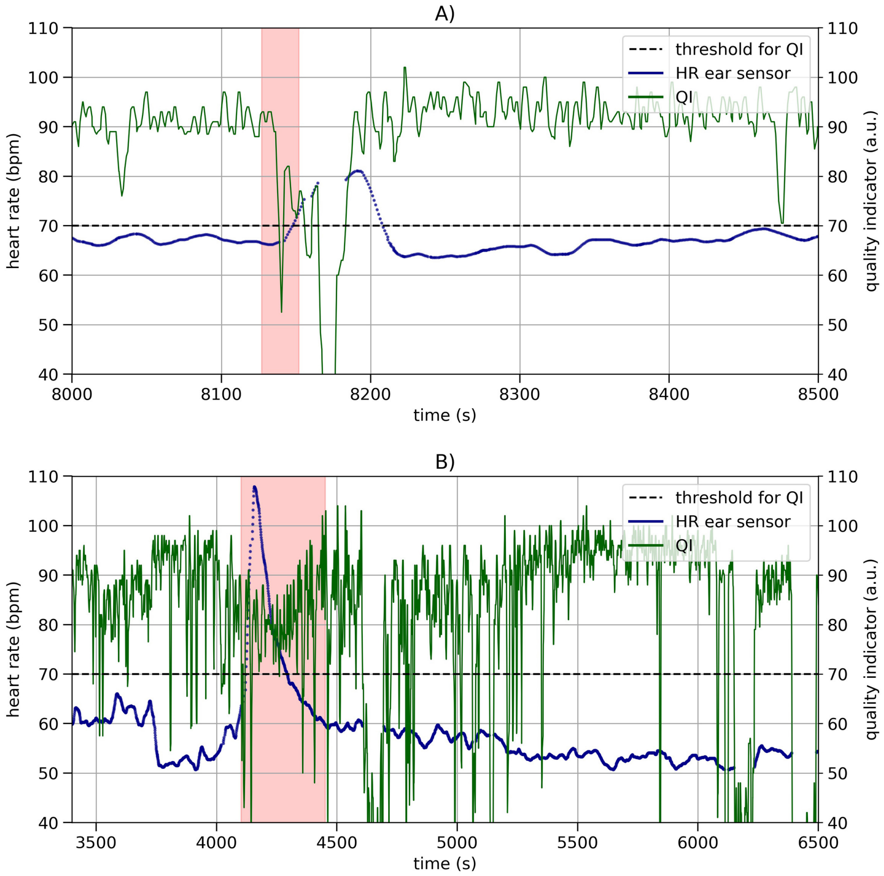

Figure S2. Plot A): An example of an epileptic seizure, marked in red, during which data was removed due to insufficient quality. Note the heart rate values reappearing at a higher level shortly after the seizure. Plot B): An example of an epileptic seizure, marked in red, during which the quality of the measurement remained sufficiently high to obtain reliable heart rate values.
